# Factors influencing oncologists’ prescribing hormonal therapy in women with breast cancer: a qualitative study in Córdoba, Argentina

**DOI:** 10.1186/s12939-019-0936-z

**Published:** 2019-02-18

**Authors:** Yolanda Eraso

**Affiliations:** School of Social Professions, London Metropolitan University, 166-220 Holloway Road, London, N7 8DB UK

**Keywords:** Breast cancer, Hormonal therapy, Oncologists’ prescribing, Health system provider, Gender, Inequalities

## Abstract

**Background:**

Hormonal therapy is an integral component for breast cancer treatment in women with oestrogen receptor positive tumours in early-stage and advanced cases of the disease. Little is known about what factors influence oncologists’ prescribing practices, especially non-biological factors, although this information may have important implications for understanding inequalities in health care quality and outcomes. This paper presents findings from research on factors influencing oncologists’ prescribing hormonal therapy for women with early and advanced cases of breast cancer in the city of Córdoba, Argentina.

**Methods:**

A qualitative study using in-depth, semi-structured interviews with 16 oncologists was conducted. A stratified purposive sampling was used to recruit female and male participants and working at 3 health subsystems (private, social security, public). Data was analysed using the Framework approach.

**Results:**

According to the respondents, factors influencing prescribing practices of hormonal therapy are varied. Women’s socio-economic status (poverty and wealth) and their level of health literacy can affect oncologists’ prescribing practices. Overall, in comparison to male, female oncologists reported more awareness of patients’ needs, more involvement in communicating drug side-effects, and in offering treatment options in private health settings. The 3 health subsystems provided a differential access to drugs and lines of hormonal treatment, which ranged from a limited availability in the public sector, to administrative restrictions imposed by the social security system, and to a lesser extent, the private sector. This happened in the backdrop of national legislation covering oncological treatments and drugs free of charge.

**Conclusions:**

Addressing prescribing practices for hormonal therapy as a distinct type of breast cancer treatment (chronic care) is fundamental in the understanding of breast cancer care and can shed light on inequalities in treatments. Identifying the underlying care gaps in the prescription of hormonal therapy can help in the design of tailored interventions.

**Electronic supplementary material:**

The online version of this article (10.1186/s12939-019-0936-z) contains supplementary material, which is available to authorized users.

## Background

Breast cancer (BC) is the most common cancer in women worldwide. According to WHO, the burden of BC is increasing in less developed countries where mortality rates are higher due to late diagnosis and lack of treatment facilities [1]. In Argentina, approximately 30–40% of BCs are diagnosed at advanced stages of the disease (stages III and IV) [2], and the survival rate of BC is 68.2%, below the 85% considered an international benchmark [3]. In addition, inequalities in diagnosis are observed within the different health subsystems whereby, according to RITA hospital database, private centres detect most cases at clinical stages 0, I and II, whilst public hospitals do so at stages III and IV [4] (p.36).

Treatment developments in the last decades, in particular hormonal therapy (HT) for women with oestrogen positive receptors, which accounts for ~ 70% of all BC cases, has proven effective in reducing the risk of recurrence and in extending survival from the disease: in postmenopausal women, 5 years of the anti-oestrogen drug Tamoxifen (TAM) reduces the risk of recurrence by about a half and mortality by about 30%, and Aromatase Inhibitors (AI) reduce recurrence by about two-thirds and mortality rate by around 40%, during 10 years after initiation of treatment [5, 6].

In Argentina, BC is the cancer with the highest incidence considering both sexes, followed by colorectum and prostate cancer. According to GLOBOCAN (2018) [7] the (ASR) incidence is 73, and (ASR) mortality is 18 cases × 100.000. Although mortality rates for BC in Argentina have followed a decreasing trend since 1997 [8] (p.31), it still occupies the second highest in South America after Uruguay.

In the province of Córdoba, the second most populated province with 3.308.876 inhabitants in the last census (2010) [9], BC has an (ASR) incidence of 65.8 and mortality of 21.4 × 100.000, whereas in the capital city both rates are higher 77.1 and 23.7 respectively [10]. According to epidemiological regional data collected by the National Institute of Cancer (2016), Córdoba is located in the Centre Region - alongside Buenos Aires, Entre Ríos, Santa Fe and the city of Buenos Aires (Federal capital) - a region that has concentrated a higher BC mortality rate (18.1) than the national average (17.4) [11]. Socio-demographic data for the province of Córdoba indicates that the population group most affected by BC (65+) is 23.4% of the population in 2016, and female life expectancy, according to the last census (2010), is 79.2 years, in both cases showing higher values than that for Argentina [12]. Also, an 8.7% of the population of Córdoba has unmet basic needs (lower than national level at 12,5%), and 1.3% of women are classified as illiterate (lower than national average) [12]. However, a recent ecology study on sociodemographic determinants associated to the spatial distribution of BC in the province, concluded that urbanisation was inversely associated to BC incidence, whereas deprivation (measured as index of unmet basic needs per households) showed a direct relationship [13]. Other indicators associated to BC such as a good supply of health services in the capital (see Study setting below) that has traditionally served the demand of nearby provinces, two University teaching hospitals with specialisation in oncology, together with an ageing female population and a higher than average BC mortality rate make Córdoba a relevant case to explore. Scholars have long emphasised the fragmented nature of the Argentinian health system, which comprises three main subsystems (public, private, and social security) with scarce synergy amongst them. Deficiencies in cancer control have been observed not only in terms of providers and resource management [14–16], but also in terms of the lack of a National Cancer Programme [17, 18], and inequalities in access to diagnosis and treatments [19–21]. The health system in Córdoba is broadly organised as follows: The majority of the working-age population has the social security health insurance system (*Obra Social*), each insurance plan being organised according to the occupation of the beneficiary and are administered by different workers unions. Within this subsystem are also the provincial insurance plans for civil servants, and a Comprehensive Medical Assistance Program for retired people (*Programa de Asistencia Médica Integral*, PAMI). A second group of high-income earners have private health insurance (*Medicina pre-paga*) offered by employers or contracted on an individual basis. Finally, the public sector subsystem, for uninsured people, is offered free of charge and financed with resources from the provincial budget and national funds for specific health programmes. According to data from 2010, 69% of women were insured by the social security (49.73%), private health (17.44%), or state plan (1.83%); whilst a 31% were uninsured [22].

Oncological treatments and cancer drugs approved by national protocols are covered free of charge in Argentina through the compulsory medical programme (*Programa Médico Obligatorio* - PMO) [23], which applies to all health insurers including the public sector that provides drugs (approved by a protocol) through provincial health ministries. Despite these provisions to guarantee free access to oncological drugs, a study based on a survey of oncologists prescribing adjuvant BC treatment in 2008, concluded that there were considerable disparities between what oncologists thought was an ideal treatment and what they actually could prescribe to patients due to different restrictions. Revealingly, only 40% were satisfied with the hormonal treatment given [24]. This raises questions about accessibility to treatment in a context of, in principle, universal drug coverage.

Inequalities related to BC treatments outcomes have been extensively documented in the US and European countries, where modifiable social factors such as ethnicity, literacy, doctor-patient’s communication, socio-economic status (SES), drug accessibility, and health system provider amongst others, have been variously identified as drivers for different outcomes between affluent and disadvantaged groups [25]. Understanding how these different factors interact in complex ways is relevant to ensure an equitable access to HT treatment, especially if we consider that the benefits associated with HT imply a long-term process, as recent guidelines recommend a 10-year course of therapy. The significance of the latter has led authors, such as Beryl and others [26], to differentiate patients’ decision-making process into acute treatments (surgery, radiotherapy and chemotherapy) and chronic care (HT), because of the irreversible/reversable nature of the decision, and the passive/active role of treatment administration respectively. Much of the quantitative and qualitative research on HT treatments have focused on patients’ perceptions and experiences in relation to adherence to treatments. Yet there is a dearth of analysis focusing on what factors influence oncologists’ prescribing practices, the challenges they face and how they overcome them. In addition, oncologists’ gender is a variable that has rarely been explored in terms of interactions with patients and decision-making patterns, although it is known from studies on physicians more broadly, that female doctors tend to deliver a more patient-centred style of communication [27].

Hence, to help fill this gap in the literature, the aim of this study was to explore the factors influencing oncologists’ prescribing practices of HT for women with early and advanced BC in the city of Córdoba. The research questions the study explored were: 1) What biological and non-biological factors influence oncologists prescribing HT?; 2) How does the oncologist’s gender affect HT prescribing practices?; 3) How does the health system provider affect oncologists' prescribing practices?

## Methods

### Study setting

The study was undertaken in the city of Córdoba, capital of the homonymous province, where cancer treatment is provided by different specialised services available through the three health subsystems. These comprise the following: 1) the institute of oncology (public sector) concentrates services on clinical oncology and radiotherapy for the province, and provides oncological drugs to patients there assisted and by referral from other provincial/university hospitals. 2) oncology services provided by 4 private hospitals and approximately 14 clinics with various levels of complexity that have contracts with the different social security and private health insurance plans. The difference between these providers is that the latter tend to offer the most prestigious, state-of-the-art hospitals and clinics, many of which are owned by the providers themselves. It is also worth emphasising that the patients assisted in each of these subsystems often move across services making the boundaries between them not so demarcated. On the one hand, the public institution grants access to any person free of charge, so a patient in the private/social insurance sector can seek a second opinion for their treatment in the public sector or become a service user when they have lost their jobs and their insurance plans. On the other, a private clinic that predominantly receives users from  the social security system can also offer services to a handful of private health insurers. What is clearer, is that uninsured patients only have access to the public health system. For this study, the main public oncology institute, three private clinics (mostly social security), and three private hospitals (mostly private health) were purposively selected to encompass the three health subsystems based on the list of oncology services available at the provincial social security Web site [28] and through communication with hospitals directors and heads of oncology services (See Table 1).

**Table 1:** Study Participants (*n* = 16)

Gender	Participants (*n*)
Female	7
Male	9
Profession	Participants (*n*)
Clinical Oncologist	13
Gynaecologic Oncologist	2
Radiation Oncologist	1
No. Years Oncology practice	Participants (*n*)
0˗5	2
6˗10	3
11˗20	4
21˗30	4
More than 30	3
Health System Provider	Participants (*n*)
Public health	6
Social Security (Obra social)	5
Private health insurance (Pre-paga)	5

### Study design

A qualitative study design was developed to obtain insight from oncologists who prescibe  HT in the city of Córdoba. The study employed individual semi-structured in-depth interviews to explore biological and non-biological factors, structural factors (health system) and personal characteristics (gender) associated with HT decision-making and prescribing practices as perceived by oncologists themselves. Adopting a thematic analysis approach (Framework method) allowed for the identification of these pre-selected themes as well as emergent themes generated from the data. Moreover, a qualitative approach can offer depth and detail on the experiences of oncologists’ prescribing practices that could elicit the development of complementary quantitative studies.

### Data collection

For data collection, a stratified purposeful sample was used to obtain representatives of male/female oncologists working at the different health subsystems. Because this study wanted to explore the perspectives of oncologists in prescribing HT, social aspects such as gender, and health service provider – here used as a proxy to class – were considered as relevant variables within the sample. According to Patton [29] (p. 240), ‘the purpose of a stratified purposeful sample is to capture major variations rather than to identify a common core, although the latter may also emerge in the analysis. Each of the strata would constitute a fairly homogeneous sample.’ A total of 16 individual semi-structured in-depth interviews were conducted with oncologists who regularly prescribed HT to women in adjuvant and metastatic stages of BC disease.

Participants were identified and recruited through initial contact with oncologists located at the 3 health subsystems (1 director of hospital, 2 heads of services). All oncologists interviewed were asked to further identify other potential participants with personal contacts, to whom the researcher contacted via telephone and email. The sample size was initially planned for around 20 participants and it followed  the concept of ‘information power’ [30] whereby the narrow aim of the study, the specificity of the participants included and the theoretical background (health system and gender) would offer sufficient focus for the interviews. 18 oncologists were approached and only 16 participated (1 interested but did not provide interview dates; 1 non-respondent). All interviews were conducted in Spanish by the author, who is a native speaker, and is familiar with health studies research on BC endocrine treatment and its use amongst eligible patients. Interviews were digitally recorded at participants’ consulting rooms and hospital offices during July 2016, and lasted between 37 and 101 min.

An interview question guide was developed prior to the recruitment process, which included a set of questions for three different themes related to HT: prescribing, adherence, and novel hormonal therapies. In this article, only results on prescribing are presented, and the question guide is available in Additional file 1.

### Study sample

Participants for this study were 9 male and 7 female doctors, with specialisation in clinical oncology, radiation oncology, and gynaecological oncology. All with experience in prescribing HT, and with a wide breath of years in BC practice: For women, the median number of practising years was 16.4 (range: 5–40); and for men 21.3 (range: 5–54). This reflects the late feminisation of the oncology profession in the last 40 years.

Regarding the health service provider, participants worked at different settings (clinics, hospitals, and institutes) corresponding to the 3 health subsystems. For this study, the identification given to each of the 3 subsystems has followed, in the case of private/social security, the main type of population that the service assisted. Finally, it is also common for doctors in Argentina to work at different institutions, and within this sample, a few oncologists worked simultaneously in two different subsystems (one worked in the private and public sector, and three did so at the social security and the public sector). In this sense, the questions were focused on their perceptions and working experience of the specific setting where the interview took place.

After preliminary analysis of the data it was considered that sufficient information power regarding relevant patterns of prescribing practices was obtained before completing the 16 interviews. Characteristics of the sample are provided in Table 1.

### Data analysis.

All interviews were recorded with participants’ consent, and transcribed verbatim by a research assistant with experience in qualitative data management. The author subsequently double checked the transcripts with the recordings and translated it into English. Framework analysis was used to analyse the data where a combined approached was adopted, first, through a deductive process based on the literature that informed the research questions and secondly, through and inductive process based on participants’ accounts [31]. Data were analysed following the five methodological steps of the framework analysis: familiarisation with the data, identification of a thematic framework, indexing, charting, and mapping and interpretation of themes [32]. Data was entered into a case chart for each respondent were notes and extracts of relevant passages were included for all identified themes (see Additional file 2 for an example of the case chart used). This allowed further identification of patterns and associations during the mapping and interpretation process of the similarities and differences in relation to gender and the health system provider.

Data interpretation is reported here by using relevant verbatim quotes to illustrate.

### Quality assurance

In order to increase the internal validity of the data collected, member checking was systematically used during the interview and through the presentation of a summary of the information collected to each participant at the end. This allowed the investigator to paraphrase answers provided by each respondent, and to ensure understanding and accuracy in the presentation of ideas.

To ensure reliability and confirmability of data collected and analysed by a single researcher, the process of indexing (using textual codes) and charting was conducted in two different stages. First, indexing and charting was developed by using the Spanish transcript. After translation into English, a second round of indexing and charting took place fourth months after the first one. The recoding (crosschecking) of the two versions enhanced the process of refining themes and subthemes, and ensured the elimination of ambiguity of terms and lack of clarity, as well as the researcher’s subjectivity and bias.

## Results

Four core themes emerged from the analysis of the interviews: Biological and clinical factors; treatment guidelines; patients’ socio-economic status; and the health-care provider (access to drugs). Several subthemes are clustered under each core theme.

### Biological and clinical factors

#### Tumour biology and age

The oncologists interviewed stated that the main indicator for prescribing HT was the presence of oestrogen receptors (ER+) alongside other markers such as progesterone values, HER-2, and proliferation marker Ki-67. These corresponded to the standard assessment of predictive factors, with the exception of multi-gene profiling assay, which due to costs, was not readily available. Other indicators considered were, disease stage: HT is prescribed for primary (early) stage, and locally advanced BC where treatment aims to reduce the risk of recurrence and is considered ‘curative’, and in advanced cases (metastasis) with low tumour volume, where the role of HT is to extend years of life. They also referred to prognostic factors such as nodal status, especially for indication of chemotherapy in young women before initiation of HT. Finally, the age of the patient (pre or post menopause), and women < 35 years-old who were considered as ‘higher risk’ patients. Oncologists were asked to describe in broad terms the indication of HT for BC, and some described it this way:

With the immunohistochemistry report that expresses the hormonal receptors, we have a predictive factor of response. From there, we select which will be the therapeutic tool of the hormonal therapy directed to that disease: anti-oestrogens, aromatase inhibitors. If pre-menopause, one therapeutic strategy will be provided for them, which is Tamoxifen; for post-menopause it is possible to offer Tamoxifen and Aromatase Inhibitors which is not possible to give in pre-menopausal women. […] Either because of risk issues related to the disease or because one must weight comorbidities, both will direct the selection of the therapeutics, the hormone therapy more appropriated for each case (09, male, social security).

Here [public hospital] we use the same as it is used in the standard protocols whether national or international. We use Tamoxifen as first line only as long as receptors are positive in pre and postmenopausal women. Then we will see in the postmenopausal, depending on age, if we need to use an Aromatase Inhibitor, such as Anastrazole or Letrozole (02, female, public sector).

Whilst there were no relevant differences amongst respondents in terms of the biological factors considered for prescribing HT, most participants remarked on the many biological considerations involved.

It is difficult to resume in few words because breast cancer is one of the largest chapters in cancer treatment (01, female, social security).

You are asking a question that is too general, which demands a more specific answer. All depends on the type of cancer, the receptors, the age of patients, […] if you say a woman with 80 years-old with a conserving surgery, has receptors highly positive, it is a small tumour, early stage, or doesn’t have risk factors, we can go with conserving surgery, radiotherapy and hormone therapy, yes, we can do [..] But if you have positive hormonal receptors with a HER-2+++, is totally different (02, female, public sector).

#### Co-morbidities

Oncologist also expressed that patients’ clinical morbidities at the start of the treatment alongside known drug toxicities were important factors in the decision-making process. For example, women with varicose veins and vascular pathology were not prescribed TAM, and in patients with osteoporosis, AIs were contraindicated. Also, because AIs inhibit the conversion of androgens to oestrogens in peripheral adipose tissue of postmenopausal women, in the case of obese patients, AI-Letrozole was preferred to AI-Anastrazole because of its treatment efficacy. Clinical morbidity and drug toxicity, added to tumour biology, led oncologists to develop a disease narrative of individual, each case scenario. As these oncologists described it:

Each patient is unique. To one we make one scheme, to another other (03, female, public sector).

The therapeutic guidelines start to personalise for each case. From the general recommendation, one has to personalise, for each case, for each patient by name and surname […] Women with the same disease can have different therapies because each of them has its own biological history (09, male, social security).

### Treatment guidelines

In general terms, the protocols in use followed the clinical practice guidelines from the American Society of Clinical Oncology (ASCO), National Comprehensive Cancer Network (NCCN), the European Society for Medical Oncology (ESMO), and St Gallen International Breast Cancer Consensus Conference. These guidelines have largely informed the provincial protocol elaborated by the Córdoba Association of Clinical Oncologists [33], whose recommendations are harmonised with the largest provincial social security provider (APPROS) that mainly covered workers in the public health sector. Social security and private health providers also used international guidelines, however, they covered different drugs in their respective insurance plans.

#### Experience

Some oncologists, however, provided a more nuanced account about the strict attachment to these guidelines, in particular those with more years of practice, who expressed some reserve based on experience, a preference for meta-analysis, and familiarity with a drug. These were identified in both male and female participants and in all service providers:

I hate the guidelines ... here there is a great use of guidelines, because doctors follow the guidelines and they don’t read. […] I only follow meta-analysis and all RCT with more than 10 years follow-up (04, female, social security).

One has acquired a certain experience that allows you to continue practising  with good parameters and with acceptable results. Even though you are not using the latest trend! […] Because we have seen many drugs, things and projects that have disappeared because it has been proven that they were not so useful as it was initially thought (11, male, private health insurance).

There are ‘grey’ situations, where the experience and opinion of each doctor sometimes counts more than the guidelines, isn’t it? [..] In general the guidelines contemplate all the options, if one sees the guidelines they are not categoric because they are guidelines precisely, are consensus of experts based in the evidence (10, male, social security).

In another case, and although the ASCO (2016) guidelines and provincial protocol recommended the use of TAM or AI for early stage ER+ postmenopausal women, an oncologist expressed,

There is a tendency to use Aromatase Inhibitors in postmenopausal patients over TAM. In my case no, I prefer TAM and [Aromatase] Inhibitors I only use them in those patients that could benefit due to the risk of disease recurrence (08, male, social security).

#### Uncertainty

Some oncologists referred to the constant updating of guidelines, and the need to follow-up the new consensus achieved by expert meetings. One oncologist described this as:

What I am using now [2016], maybe it will be discussed in March 2017 (01, female, social security)

Another oncologist tried to reflect on the reason why the guidelines for a drug like TAM has changed so much over time:

When I graduated, we used to give Tamoxifen for life. Later, it was for 5 years, then for 3 years, then it returned to 5 years, and now is 10 years. There must be a bias in all this… There is more money to research certain things than others, which are the ones that the industry is interested in, and they allocate more money for that. I mean, not everything is researched with the same allocation of funds… (05, male, private health insurance).

### Patients’ socio-economic status

#### Socio-economic position and life circumstances

In making a treatment decision, oncologists expressed that patients’ socio-economic and socio-environmental factors affected their treatment options, and believed that their decisions were made in the patient’s interest. This was mentioned in two scenarios, when deciding amongst all treatments available, especially chemotherapy; and when prescribing a particular drug (molecules) corresponding to a hormonal line.

We need to bear in mind the [social] conditions and the reality of patients. Sometimes we assist  patients of very poor social condition, that live in total deprivation, overcrowded, so one needs to consider these issues. How will you prescribe chemotherapy, that can lead to neutropenia, leokopenia if you know that the patient won’t have the support that she needs? […] In those cases, we discuss within the team, but we give hormonal therapy instead (14, male, public sector).

A poor woman, who lives isolated in a rural area, we cannot use new molecules due to the toxicity (neutropenia, diarrhoea). They need to be near a hospital! (16, female, private health insurance).

Male oncologists in the public sector tended to see socio-economic limitations as less problematic for the prescription of HT in comparison to the weekly hospital visits, transport costs, and health risks from chemotherapy:

The hormonal treatment is totally manageable. The patient that uses TAM, the Ministry gives her 2/3 boxes, so for 3 months she doesn’t come, and you realise in 3 months when she comes back to ask for the renewal of the prescription (14, male, public sector).

Female oncologists, however, considered HT prescription as being sometimes problematic when low health literacy was involved:

Many times it has happened to us that we give them the medication and they return in 3 months, and they say ‘I took it once Dr.’, this means that often they don’t understand the treatment even when you write it down for them, and even when they know how to read, they don’t understand it (13, female, public sector).

Valuing the patient’s quality of life, women’s daily activities and responsibilities, emerged as important factors only for some female oncologists in the private sector. For these doctors, assessing clinical factors was considered in tandem with social ones, thus both being weighed in prescribing a particular hormonal line:

The patient decides. We consult all with the patient. Sometimes they have a very active life, a work life, etc. and we recommend a therapeutic line based on toxicity. We mention side effects, such as fatigue [aromatase inhibitors], and discuss options with them (16, female, private health insurance).

#### Discussing treatment options

The discussion of treatment options with patients elicited different perceptions. One respondent reflected with a comparison with the shared decision-making model that prevails in US and European contexts in relation to patients’ engagement, information sharing and participation in treatment decisions.

In Argentina and Latin America there is a more paternalistic attitude, that the population knows. When one presents this [treatment options] to patients and when we tell them, “if you don’t want to do anything, it is your right”, they say “no, doctor, if you are asking me, I will do it”. […] The patient knows everything [treatments and side effects] because one tells them, and they need to sign an informed consent. We do have patients that reject treatment, although not frequently (02, female, public sector).

Male oncologists in particular, saw the issue of discussing treatment options with patients as a process given by the whole therapeutic spectrum, as information-sharing and patient’s decision regarding acute treatment (surgery, radiotherapy and chemotherapy). However, in the case of HT, male oncologists tended to consider it as a benign, non-toxic treatment in comparison to the acute ones, hence they did not contemplate the need to discuss with women the available options. As one participant explained, the decision of which hormonal line can be prescribed needed to be taken by the specialist after assessing a woman’s clinical condition (comorbidities).

In hormone therapy one evaluates the risk of the patient. Today I saw a patient with phlebitis, this makes her no eligible to treatment with Tam. The election [aromatase inhibitor] was mine! I proposed what I considered to be more effective for her. But with chemotherapy no. Because we have many more adverse effects, from the aggressiveness for health, to what affects emotions, psychological, aesthetics and psychospiritual wellbeing for the patient. There, yes, it is possible to discuss, and the patient participates actively in the election of therapeutics or the rejection of the therapeutics. In hormonal therapy no, because fortunately they are very well tolerated (09, male, social security).

#### Health literacy

Most respondents perceived patients’ health literacy as being stratified by social class. They largely concurred about a clear distinction of patients’ behaviour across the three health subsystems in terms of passive/active interaction when treatment was communicated, and patients’ expectations during the encounter.

Female oncologists in the public sector acknowledged that for patients with low health literacy, good communication and information, contention and encouragement since the initiation of chemotherapy for advanced cases (mostly observed in the public sector) was fundamental for their ability to prescribe HT. A range of social support offered by the provincial Ministries and a cancer NGO including transport costs, education, and workshops, secured patients’ contention and prescription of HT as chronic treatment.

Breast cancer is a chronic disease, so you need to give tools to the person so she can be able to do something with her life. So, if she hasn’t finished school… or another activity is the prevention of lymphedema, so we give women the tools for them to do exercise for free, because they don’t have money (02, female, public sector).

Male oncologists considered that the most deprived and the most affluent patients could pose a ‘burden’ during consultations for treatment prescription due to the former not asking any questions at all, and the latter asking too many questions. The following quotes exemplify how they experienced these interactions:

The lower the socio-economic position, the higher the tendency to accept what the state offers them. The higher the socio-economic level, the more demanding are the individuals in relation to the health insurer, institutional services, doctors and the time they give to them (10, male, social security).

If the doctor tells them what to do, they go and do it. They generate a huge burden on us because they don’t ask you anything, you see them totally surrendered to what you tell them (14, male, public sector).

Dr Google is an important colleague we have! There is a lot of information out there but there is a need to organise it. People read more, are more informed, and Dr Google would be the first problem and Dr Neighbour would be the second one. A neighbour that tells her [patient] that she has an aunt, or a cousin that has the same [disease] like her but she was prescribed another thing... (05, male, private health insurance)

We need to give them [patients] the information why… the truth is that people ask for explanations. On the other hand, today they are very informed by the internet, so they keep abreast. […] Women ask why this, why the other, which benefits and so on… Here we are very nosy, you know that we are an exigent society (07, male private health insurance).

### The health-care provider (access to drugs)

Oncologists were asked about the main issue that affected prescribing practices, and the one that the majority identified was the different access to drugs. They referred that each provider in the public, social security and private sector covered different drugs, and therefore, allowed them to prescribe different lines of hormonal treatments. Some of the challenges that respondents identified for each of the providers are presented below:

#### Prescribing outside the protocol

Oncologists working in the public sector spoke about the limitations in accessing drugs that are outside the protocol, which is the list of drugs that are provided by the Ministry of Health of the province.

In the public sector is totally different from the [HT] that is used in the private sector. [...] We have fewer resources and they have imposed to us a protocol that is very poor (14, male, public sector).

We usually do Tamoxifen in adjuvancy, that is what we’ve got faster in terms of approval by the Ministry. […] We use more Tamoxifen in postmenopausal. That will be perhaps the difference with other institutions. Yes, due to cost and accessibility perhaps the [Aromatase] Inhibitors are more used in private settings. In general, we use Inhibitors as a second line, or intolerance to Tamoxifen (12, female, public sector).

At the same time, they referred that they did have access to other drugs outside the protocol through a request to the National Ministry of Social Development, but they found the procedure very onerous. The comments were that it was bureaucratic in terms of the paperwork involved, time-consuming for the doctor, and tiring for the patient. Some of them described this as follows:

We can have access to other type of medications, but the procedure is a bit bothersome for the doctors and also for the patient, because it is too much paperwork involved, and the delays is around 60/90 days. But we have access to other new medications like Palbociclib or others like Fulvestrant, that we don’t have in our protocol.

[…]

For a medication so common [Fulvestrant] that we normally ask in the private sector or for patient in the social security, to have to fill in a thousand forms [in the public sector] and every 3 months to send all the clinical records that demonstrate that [the patient] is responding to treatment… We have overloaded with patients, and many appointments and, on top of it, the bureaucratic side … it tires you (14, male, public sector).

#### Dealing with social security and private insurance plans

For these providers there was, according to participants, a larger range of drugs available, yet the accessibility to these drugs varied according to the provider. Those who offered newly available and expensive drugs also attempted to restrict access, through a highly bureaucratic process that involved patients and oncologists. Most oncologists were able to name specific drugs as accessible or not in the main social security and private insurance plans. They also mentioned certain, more expensive drugs (Exemestane, Fulvestrant, Goserelin) that were covered in principle, but doctors needed to justify why the drug has been recommended. As many oncologists working in this subsector concurred:

Treatment is according to patient and her social insurance plan. […] In adjuvancy there is a strong tendency to use Anastrozole in postmenopause because a study gave 2.5% of benefit. Well, I’ll see… because for the insurance plan is much more expensive Anastrazole and the differences are very little. If I see that they [social security] are not going to give the medication, or they will delay it, or for the patient is a cost, I am sure I will use Tamoxifen (04, female, social security).

What we observe is that the insurer won’t reject up front a drug prescribed because it doesn’t want to enter into conflict with the patient  […] and it starts, in a subtle way, to put obstacles in the way of the provision, avoiding to say up-front ‘I won’t cover it’, but the patient enters in a complicated process with paperwork and medical check-ups that wears the patient out in the request of the drug (09, male, social security).

For a [drug] request, we have to deal with each social security plan to see whether they will accept it or not, some ask for more requirements, other less … It is complex. It implies for doctors an onerous task, because of paperwork, forms, reports, engagement with auditors (10, male, social security).

However, the request of drugs in the private health insurance was not perceived as a problem.

In general, it is standardised in the social security and private health insurance to fill in a questionnaire for the first time that one prescribes to a patient: to state diagnosis, age of the patient, the objective why a drug is requested, for how long. We make a kind of protocol that we always do for the first time, but it is not that they oblige us to put something special. It is something standard […] an initial auditory. […] later on, every time we use a new medication, they ask us to fill in a form explaining why we change. But no more than that (06, male, private health insurance).

We are here within the private sector and we have greater accessibility, but we know that in the public sector that is a bit more limited (07, male, private health insurance).

## Discussion

This qualitative study explored factors associated with oncologists’ prescribing practices of HT for women with oestrogen positive BC. To my knowledge, is the first qualitative study on this topic conducted in Córdoba, Argentina. HT is highly standardised in Western medicine through consensus guidelines elaborated by professional associations in the US and Europe, based on evidence-based clinical studies on populations. Whilst adherence to these guidelines, in the sample studied, seems to offer a consistent approach to treatment decisions, patient-specific factors such as tumour biology, clinical morbidity, drug toxicity, and tumour resistance allowed oncologist to develop an individual-case approach. The role of experience in the older generation also offered oncologists a way to exert choice and regain clinical judgment in relation to the standardisation imposed by guidelines. In addition, two other factors regarding guidelines’ applicability explored by the literature have been expressed by the respondents: firstly, the local resource implications (drug availability), which requires adaptability and consensus by the team of specialists, as observed by a study on cytotoxic drugs prescribing in the city of Rosario (Argentina) [34]. Secondly, the constant following of updates to guidelines to keep peace with emerging data [35].

More importantly, a range of non-biological factors appears to add complexity to the way the prescribing of treatment is formulated. Factors associated with patients’ SES was an influencing component in decision-making observed in this study, comprising three interrelated elements, socio-economic position, discussing treatment options, and health literacy. Although patients’ SES has not been specifically analysed in Argentina, a report exploring inequalities in access to drugs in the public sector pointed to the existence of ‘a cultural profile of the public sector patient and the health professionals that assist them, [as characterised by] a tendency to accept the disease and its associated problems with resignation, including the limitations of the care services’ [36] (n/p).

Overall, this study has shown that SES factors played a role in prescribing HT treatment, and that there were different approaches observed according to oncologists’ gender. Even when most respondents acknowledged the difficulties that women living in very deprived conditions can pose to chemotherapy, male tended to consider HT as a more acceptable treatment ‘for all social classes’ (14, male public sector). This approach is, however, problematic, as it associates HT with ‘taking a pill’, without considering women’s self-management skills and understanding of the treatment. Health literacy, i.e. having the skills, knowledge and confidence to take decisions on medical instructions, is largely associated with socio-economic circumstances, whereby the most deprived groups are more likely to have low health literacy [37]. However, as shown in this study, highly educated women could also have limited health literacy − which is understandable considering the complexity associated with treatments regimes in HT − and therefore, they were perceived by male oncologists as often demanding and asking too many questions. There are only a handful of studies that have measured health literacy in specific contexts in Argentina [38, 39], but there are  none for cancer. This is an area that will require more research to find out the implications it may have for health inequalities: a study has demonstrated that BC oncologists spend more time in consultations with highly educated patients than with low-income, less educated ones [40], whilst other studies, similarly to what have been identified here, have shown that most deprived patients have greatest information needs and support in understanding their disease and treatment [41, 42].

Previous research has noted that discussion of BC treatment choices between oncologists and patients has been associated with better health outcomes [43], and more shared decision-making in adjuvant therapy was associated with greater treatment satisfaction [44]. Whilst the process of shared decision-making is not incorporated into Argentina’s health policy, where a rather ‘paternalistic model’ prevails with a right to informed consent [45], by considering oncologists’ gender as a variable, this qualitative study has shed light into how gendered power dynamics may affect the process of prescribing in different ways: On the one hand, female oncologists were more considered of the different side-effects that drugs can have in women’s quality of life and so they were more prone to consider women’s life circumstances and allow more educated women to share their views and discuss treatment options. On the other, the data collected suggest that prescribing practices in male oncologists seem not to be influenced by these considerations. Furthermore, male perceptions of both low and upper-class women can inadvertently normalise issues regarding women’s needs and understanding of treatment options, foster a sense of adherence to treatment initiation which may have implications for the amount of health information provided. Moreover, this can also have further implications for effective self-management, where oncologists and coordinated support from the health team is needed for patients to make treatment decisions and manage chronic conditions such as BC [26]. Hence, further research is needed on health information interventions such as patient decision aids, as evidenced by a Cochrane review [46], designed to meet the information needs of the different group of patients, according to their level of health literacy.

The fragmented nature of the Argentinian health system has often been identified as a cause of main health inequalities, between and within provinces, including cancer diagnosis and treatment [47] as well as access to oncological drugs [36]. The perspective of oncologists indicates that HT is strongly contingent on the health system providers, which limit the line of treatments (drugs) available from approximately 2 in the public sector to 7 in the private health insurance. Although oncologists tended to view these differences in the adjuvant setting as non-fundamental in terms of a reported 2–3% variation in drug effectiveness, in cases of metastatic BC the differences between lines of treatments and health outcomes can be more marked in overall survival as shown in a recent review [48]. New drugs improving the action of anti-oestrogens as well as different generations of anti-oestrogens (SERD) and AIs open different sequence and combinations of treatments (Everolimus with Exemestane or TAM; Palbociclib with Letrozole or Fulvestrant). Precisely some of these targeted drugs are the ones that appear as more challenging to access in the social security and the public sector.

The recurrent mentioning of bureaucracy and paperwork involved in accessing drugs that are not covered within the Provincial/National protocol menu (public sector), or that are covered (social security) but its access is made very difficult by insurance plans can also make the prescription of treatment dependent on both determined oncologists and patients. Participants’ responses are in line with findings from Argentinian reports whereby oncological patients in the public sector referred to delays in access to medication due to wrong prescriptions and complicated paperwork from social services, as well as from national or provincial drug suppliers [18, 36]. For insured patients, lawsuits have increasingly become an alternative way of accessing oncological medicines [49]. In the private health subsystem respondents saw the request of drugs as less challenging. This could be due to a more manageable workload for professionals, a better coverage from the insurance plan or a simpler administrative procedure from the insurer. Finally, in requesting drugs outside the protocol, oncologists needed to consider, as one of them expressed, ‘The possibility to sustain treatment in the future’ (07, male private health insurance), that is, to balance the risk of discontinuing treatment after an initial medication approval.

## Limitations

Some limitations of this study require careful consideration. Although the participants within the sample represented the three health subsystems and oncologists’ gender, it is possible that their views in relation to gender approaches to care, is not representative of all male/female oncologists working in these different settings. Also, the prescribing practices here described for the city of Córdoba cannot be generalised to Argentina, even in the presence of the same tri-partite health system. Gender notions, in particular, are informed by societal factors, which may largely differ amongst cities with different levels of community values, medical education and training, and patients’ demands and expectations of care.

## Conclusions

The findings of the study identified a number of themes expressed by oncologists in their prescribing practices of HT for BC women in Córdoba. Overall, the empirical data collected suggests that HT is largely perceived by oncologists as a ‘different type’ of treatment, which is set ‘in comparison’ to the acute ones (surgery, chemotherapy and radiation). Considering HT as a form of chronic care is fundamental to address the specificities involved in oncologists’ prescribing practices and in the understanding of HT use among breast cancer patients. The intersections of biological (tumour type, age and co-morbidities) and non-biological factors (HT guidelines, patients’ SES, women’s ability to understand HT, and access to drugs), alongside the physician’s gender and the health system provider can variously and simultaneously influence oncologists’ decision-making. Given the different lines of treatments available within HT (according to tumour type, age, co-morbidities, and prognostic factors) effective communication of medication options can pose a challenge to oncologists and may disadvantage women affected by the disease, by inhibiting their comprehension of treatments options, benefits and risks. The use of tailored interventions such as patient decision aids delivered to BC women pre or during consultation, and designed according to the perceived health literacy needs (e.g. for low-literacy groups, a web-based interactive audio-visual intervention provided at the hospital with the assistance of a nurse; for high-literacy groups, a booklet or DVD using plain language), can facilitate women’s informed decisions and make it easier for oncologists to discuss treatment options.

In this study, oncologists’ gender can be a contributory factor on treatment decision-making. Female oncologists in the private and public sector, tended to be more responsive to women’s needs, more prone to discuss treatment options and drug toxicity. More qualitative research, including patients, will contribute to advance knowledge on oncologists’ gender as a specific factor, before policy implications can be drawn.

Of significance, the fragmented health system can lead to a differential access to drugs or lines of HT in the metastatic BC setting between the public and private sector; and also to a restricted access to  drugs that are covered through the insurance plans. Health system coordination amongst the subsectors, a role assumed by the Ministry of Health of the Province, should be improved in terms of drugs accessibility if equity in access is to be achieved. Initiatives should be taken to make the provision of oncological drugs more streamlined and accessible to all service providers.

## Additional files

Additional file 1: Interview question guide. (PDF 65 kb)

Additional file 2: Case chart. (XLSX 12 kb)

## Abbreviations

AI: Aromatase inhibitors
BC: breast cancer
HT: hormonal therapy
SES: Socio-economic status
TAM: Tamoxifen
