# Supplementary material for: Factors influencing oncologists’ prescribing hormonal therapy in women with breast cancer: a qualitative study in Córdoba, Argentina
Source: Int J Equity Health. 2019 Feb 18;18:35. doi: 10.1186/s12939-019-0936-z (PMC6379997; doi:10.1186/s12939-019-0936-z)
Supplement: Supplementary file 1 — Interview question guide. (PDF 65 kb) [file 12939_2019_936_MOESM1_ESM.pdf]

Interview question guide

1. Can you describe the hormonal therapy for breast cancer you use in your clinical practice?
2. What are the main biological/clinical indicators you consider when you decide to prescribe hormonal therapy?
3. When you prescribe hormonal treatment, are you following a particular cancer guideline or protocol?
4. What are the drugs in use in your clinical practice?
5. How are decisions made regarding the different lines of treatments available for early and advanced stages? (probes: drug toxicity, duration and switching between drugs)
6. How are drugs funded in your service?
7. Are there any problems in accessing drugs in your health setting? If yes, can you talk about the main issues you encounter when prescribing drugs, and if you overcame them and how?
8. Apart from biological or clinical conditions, are there any other issues you take into account when prescribing hormonal therapy? (probes: patients' material/life circumstances, family, psychological aspects)
9. Do you discuss treatments options with your patients? If yes, what issues do you identify as most challenging when discussing treatments with patients?
